# Reviews and Book Notices

**Published:** 1882-12

**Authors:** 


### Reviews and Book Notices.

---

ARTICLE IV.—A SYSTEM OF HUMAN ANATOMY, Including its MEDICAL AND SURGICAL RELATIONS. By HARRISON ALLEN, M.D., Professor of Physiology in the University of Pennsylvania, etc., with a Chapter on Histology by E. O. SHAKESPEARE, M.D. Six sections, containing about 550 pages of letter-press, illustrated with 380 figures on 109 plates, many of which are beautifully colored. The drawings by Hermann Faber, from dissections by the author. Also 250 wood cuts in the text. $3.50 per section. Philadelphia: Henry C. Lea's Son & Co., 1882.

Each section of this work is contained in a cloth cover, rather artistic in design, and eminently adapted for use beside the cadaver, but rather inconvenient in the library. A work like this had been anticipated for some time by those aware of the rapid progress manifested of late in comparative anatomy. The best works on Human Anatomy were, in fact, rapidly growing old. The New Anatomy comes to us with unexpected features, the most remarkable of which is an interesting style. The latest discoveries of science are mentioned in its pages; and it is published in a large clear type and on the best quality of paper. However, for the sake of practitioners we would recommend the publishers to issue leather-bound copies in two or three volumes, separately, as the present style is inconvenient in the office. The part on histology is thorough, but too little of it seems original and so with the illustrations, which are almost too beautiful to be real. The same assertion would not be true of the plates of the second volume, "bones and joints," which are rather inferior in execution. But works of anatomy should be mainly illustrated by dissections of the human body, and ordinary plates cannot teach much, and should not. The best recommendations would not do credit to this work, and the student of anatomy will be aware of its merits only after a practical use of it; but it certainly fills a wide gap and will be welcomed by all. H. D. V.

ARTICLE V.—SPEECH AND ITS DEFECTS; CONSIDERED PHYSIOLOGICALLY, PATHOLOGICALLY AND REMEDIALLY. By SAMUEL O. L. POTTER, M. A., M. D., Author of "An Index of Comparative Therapeutics," etc. Lea Prize Thesis of Jefferson Medical College. 12 mo., cloth, pp. 117, $1. Philadelphia: P. Blakiston, Son & Co., 1882.

This is a remarkably interesting little book, and, as a thesis, one of great merit, especially in this country, where preliminary medical education is recognized as deficient. The subject is treated in a most interesting manner, especially as to its history; and its bibliography has not been forgotten. In the treatment of stammering and stuttering the author recommends the *motor depressants*, gelsemium, conium, physostigma, lobelia, aconite, etc. Belladonna, hyosciamus stramonium and tonics have their indications also. A number of favorite methods in the hands of renowned quacks of the present day are mentioned and the author adds combinations of consonants and vowels for exercises.

This small treatise will be welcomed by all whose vocal organs or functions are imperfect in any degree; it will repay the curiosity of any reader and forms a valuable addition to the literature of the human voice. Although without statement to this effect, it appears that the present volume was intended as one of the Health Primers published by the same firm, and which have proven a valuable departure in medical writings. H. D. V.

ARTICLE VI.—SOCIAL EQUALITY; A SHORT STUDY IN A MISSING SCIENCE. By WILLIAM HURRELL MALLOCK, Author of "Is Life Worth Living?" 12 mo., cloth, pp. 212. New York: G. P. Putnam's Sons, 1882.

This, like other writings of the well-known author, is remarkable for its agreeable style, something which is becoming scarce in our days. That the principle which is the foundation of this work, that social inequality is natural to the human character is false, is useless to argue. But the work is timely. It opposes a democratic reaction which menaces to destroy at a stroke a civilization which has been the outcome of centuries of patient work. Let the churches disappear; let the professions disappear; let the various trades follow, and give us the—say co-operative manufacture—the optimum of democracy; would life be worth living under circumstances where the fine arts could no longer exist? Mr. Mallock's book has been strongly criticised by the scientific press, probably because the work does not conform at all with scientific principles and is simply intended for the common reader, yet, we must grant that between natural selection and arch-æsthetism, between causation and spontaneity, the very foundations of science and philosophy are not at all settled, and we firmly believe that Mr. Mallock has written a timely and serviceable book, more appropriate perhaps in his country than here.

ARTICLE VII.–FISTULA, HEMORRHOIDS, PAINFUL ULCER, STRICTURE, PROLAPSUS AND OTHER DISEASES OF THE RECTUM; THEIR DIAGNOSIS AND TREATMENT. By WILLIAM HILLINGHAM, M. D., Surgeon to St. Mark's Hospital for Fistula and Other Diseases of the Rectum, etc. Fourth Revised and Enlarged Edition, with Illustrations. 8 vo., cloth, pp. 168. Philadelphia: P. Blakiston, Son & Co., 1882.

Of the few works exclusively written on this subject, this is surely one of the most valuable. We felicitate the publishers on the happy thought of reprinting it in a cheap form, which advantage every surgeon should avail himself of. Regarding the mooted question of the coincidence of fistula in ano and phthisis, the author says that such a thing is of frequent occurrence. He regards phthisis in that case as a complication of fistula, and an operation well conducted will be beneficial (he does not say curative). As to the injection of hemorrhoids with carbolic acid, although conversant with the subject, the author deprecates the injections. In forty-nine cases of cancer of the rectum treated with Chian turpentine, only two seemed somewhat benefited. Enucleation is sometimes a valuable mode of treatment. This book possesses a good deal of intrinsic value, embodying so much of the experience of a single surgeon and is a worthy addition to a subject too neglected, in view of suffering humanity.

H. D. V.

ARTICLE VIII.—CLINICAL LECTURES ON THE DISEASE OF THE URINARY ORGANS. By SIR HENRY THOMPSON. Sixth London Editon, Illustrated with 73 Wood Engravings. An 8vo. Pamphlet of 175 pages. 75 cents. Philadelphia: P. Blakiston, Son & Co., 1882.

The author says in the preface: "It may be pardonable, perhaps, to add that I have had the gratification of finding it employed as a text-book in most of the medical schools of Europe, and translated for that purpose into the French, German, Italian, Spanish and Russian languages."

After such a work has reached its sixth edition, and has been presented to the profession in such a form, it would be unpardonable to be without it; and not only the specialist and the ordinary surgeon should have it under the hand, but it is a most excellent text-book for students. Its defects, which are worth mentioning, consist in some of the lectures being of a rather old date, and the prominence given to stone in the bladder being such that other diseases are almost overlooked. Dr. Bigelow's method is favorably commented upon, but the author refuses the name "litholapaxy," and substitutes the name "lithotrity at one sitting."

ARTICLE IX.—THE NEW MATERIALISM. DICTATORIAL UTTERANCES AND THE DECLINE OF THOUGHT. By LIONEL S. BEALE, F. R. S. Pamphlet, pp. 37. London: The Victoria Institute.

The primary object of the Institute is "to investigate fully and impartially the most important questions of philosophy and science, but more especially those that bear upon the great truths revealed in Holy Scripture, with the view of reconciling any apparent discrepancies between Christianity and science." The Darwinian or Monistic philosophy has conquered the English mind with such rapidity that it is high time that men like Beale arise in the effort to start Christian philosophy from its long inanition. As the various pamphlets of the author are intended, we suppose, for Sunday-schools and for distribution among the totally ignorant in matters of science, they will impart to the readers a smattering of knowledge which should not be despised. However, the author's imperfect understanding of modern philosophy places his works beyond the reach of those who cultivate science for itself and have no time to throw away.

ARTICLE X. — CONTRIBUTION TO PRACTICAL GYNÆCOLOGY. (Illustrated.) By S. JAMES DONALDSON, M.D., Surgeon to Gynæcological Wards, Ward's Island Hospital. Part I.—PRACTICAL OBSERVATIONS UPON UTERINE DEFLECTIONS. Part II.—PRACTICAL OBSERVATIONS UPON DYSMENORRHŒA. 8vo. Pamphlet: pp. 134. New York: Trow's Printing and Bookbinding Co., 1882.

This is a funny monograph. The author challenges the world and gynæcologists, as well-armed he is, with a new pessary surmounted with a uterine stem, and pours a large amount of vituperation against the abominable practices of his contemporaries. Therein lies the practicability of his observations. Between the two we must say that we would rather follow the method of a Chicago gynæcologist and treat deflected uteri with plaster-jackets applied to the patient's back, than use the internal splint of Dr. Donaldson.

ARTICLE XI.—A MANUAL OF PRACTICAL NORMAL HISTOLOGY. By T. MITCHELL PRUDDEN, M.D., Lecturer on Normal Histology in Yale. 16 mo. Pp. 265. New York: G. P. Putnam's Sons, 1881.

This little book comes out without any allusion to microscopy, without any illustration of histological specimens, and has an appendix of 18 blank pages which the student is intended to fill with his own drawings. But for its deficiencies, it is practical enough, and resembles somewhat as many lectures as there are chapters. The merits of this book chiefly consist in the thorough description of the author's methods of mounting specimens and will prove most useful to students in that respect. Still it is doubtful whether medical students will ever learn much histology when the short curriculum is overcrowded with so many other and more essential sciences. H. D. V.

ARTICLE XII.—A SYSTEM OF SURGERY. By SAMUEL D. GROSS, M.D., LL.D. Illustrated by upwards of 1600 engravings. Sixth Edition, thoroughly revised and greatly improved. In two volumes. 8vo., half Russia. Pp. 1194–1174. Philadelphia. Henry C. Lea's Son & Co., 1882.

In a time when works on surgery are being published at a wonderful rate, it is somewhat refreshing to see the old reliable come out in a revised form for the sixth time, and it is doubtful whether it will lose its prestige as the leading text book of surgery in the land. This last edition is up to the latest progress in the subjects of which it treats. H. D. V.

ARTICLE XIII.—THE EXPERIMENTAL METHOD IN MEDICAL SCIENCE. Cartwright Lectures of 1882. By JOHN C. DALTON, M.D. 12 mo. Cloth. Pp. 108. New York: G. P. Putnam's Sons, 1882.

These three interesting lectures have already appeared in some of the journals but have received a more dignified treatment in the elegant little volume under consideration. They will be read with pleasure, not only by the profession, but by all those interested in scientific researches, although they properly belong in the domain of Medical history.

